# Alcoholism Risk Reduction in France: A Modernised Approach Related to Alcohol Misuse Disorders

**DOI:** 10.3390/ijerph111111664

**Published:** 2014-11-13

**Authors:** Georges Brousse, Patrick Bendimerad, Ingrid de Chazeron, Pierre Michel Llorca, Pascal Perney, Maurice Dematteis

**Affiliations:** 1 Service Psychiatrie et Addictologie del’Adulte CMP B, Centre Hospitalier Universitaire (CHU), Rue Montalembert, Clermont-Ferrand Cedex 01 63003, France; E-Mails: idechazeron@chu-clermontferrand.fr (I.C.); pmllorca@chu-clermontferrand.fr (P.M.L.); 2 Unité de Formation et de Recherche (UFR) Médecine, Université Clermont 1, EA 7280, Place Henri Dunant, Clermont-Ferrand F-63001, France; 3 Service de Psychiatrie, Groupe Hospitalier La Rochelle- Ré-Aunis, La Rochelle 17000, France; E-Mail: patrick.bendimerad@free.fr; 4 Service de Addictologie, Hôpital Caremeau, Nîmes F-30029, France; E-Mail: pascal.PERNEY@chu-nimes.fr; 5 Université de Montpellier 1, 5 bd, Henri IV 34000, France; 6 University of Grenoble Alpes, HP2, F-38000 Grenoble, France; E-Mail: MDematteis@chu-grenoble.fr; 7 INSERM, HP2, U1042, F-38000 Grenoble, France; 8 Department of Addiction Medicine, CHU de Grenoble, HP2, F-38000 Grenoble, France

**Keywords:** alcohol, alcoholism, alcohol-related disorders, risk reduction, abstinence, reduction

## Abstract

During many years in France, risk reduction strategies for substance abuse concerned prevention strategies in the general population or interventions near users of illicit substances. In this spirit, the reduction of consumption only concerned opiate addicts. With regard to alcohol, the prevention messages relative to controlled consumption were difficult to transmit because of the importance of this product in the culture of the country. In addition, methods of treatment of alcoholism rested on the dogma of abstinence. Several factors have recently led to an evolution in the treatment of alcohol use disorders integrating the reduction of consumption in strategies. Strategies for reducing consumption should aim for consumption below recommended thresholds (two drinks per day for women, three for the men) or, at least, in that direction. It must also be supported by pharmacotherapy and psychotherapy, which offer possibilities. Failure to manage reduction will allow the goals to be revisited and to reconsider abstinence. Finally this evolution or revolution is a new paradigm carried in particular by a pragmatic approach of the disease and new treatments. The aims of this article are to give elements of comprehension relating to the evolution of the practices in France in prevention and treatment of alcohol use disorders and in particular with regard to the reduction of consumption.

## 1. Introduction

Risk reduction is to become a public health objective through changing consumer behaviour. It is a principle in health care that seeks to reduce the risk of occurrence of health disorders resulting from the consumption of consumer products and to prevent further deterioration if such disorders are already established [1]. In France, concerning disorders related to alcohol misuse, the strategy of informing the consumer and helping the patient to recognise his pathological consumption as a “lifestyle choice” is difficult but slowly gaining a place in interventional procedures [2]. A number of explanations can illustrate these difficulties. These will be described in detail to show how risk reduction is possible in patient management for people suffering from an alcohol use disorder.

## 2. Difficulties in the Implementation of Risk Reduction for Alcohol Related Disorders

For illicit drugs, risk reduction concerns both how the product is consumed and the risks associated with it, in particular the risk of infectious disease and overdoses. Regarding alcohol use disorders, risk reduction focuses on consumption, which lies on a continuum extending from relatively safe use up to total and permanent abstinence and passing through a controlled consumption [3]. In France, as in many countries, several difficulties concerning the conduct of this discourse can be identified.

Firstly, alcohol is a legal product, widely used even though consumption decreased during the 20th century. Average consumption by the adult population (aged 15 years and over) of pure alcohol was 65 g per day in 1935 and 27 g per day in 2008. In 2008, the male population drank an average of 43 g per day and the female population drank 13 g per day [4]. Secondly, for this product, subsequent harm is often minimized because of rewarding social and cultural representations conveyed especially by the traditional and historic consumption of wine [5]. Thus, for the general population, outside of situations of high dependency, e.g., the traditional image of the widely stigmatized “drunk” [6], the awareness of risks associated with alcohol consumption make it mark with difficulty. Thus, even acute situations associated with alcohol consumption are often attributed to “bad luck” rather than to alcohol risk *perse*, which consists in a morbid rationalization [7]. Thirdly, some data in the scientific literature, widely reported by the media and producers of alcoholic beverages, highlight the beneficial effects of low consumption of alcohol and wine in particular [8].

For drug users, risk reduction is the basis of a qualitative health approach, such as the prevention of infectious risks, beginning from the first consumption. The approach to alcohol harm reduction is quantitative and qualitative, involving consumption thresholds that carry risks (quantitative) and these should be adapted to the population to which they are addressed (qualitative). In particular, one must ask whether the prevention message (stop, reduce...) to be delivered should be adapted to the target population: general population, population at risk or persons who have problems with alcohol. In reality, users and patients may accept damage reduction messages insofar as they themselves have been able to evaluate their relationship to the product and how they would like the relationship to develop. For users, this presupposes that they are able to conduct such an evaluation; for people suffering from alcohol use disorders this is doubtful, unless they have access to support. In France, for many physicians treating patients suffering from illnesses related to alcohol, harm reduction through moderating consumption is impossible because their golden rule learned at the university is total and permanent abstinence [9]. This position has recently evolved and we propose in this article to review the key points to justify this new benefits approach for patients with alcohol problems, and to put it in perspective with respect to the benefits of abstinence. We will return to the relevance of a dialogue on reducing overall consumption for the general population, which should complement the message directed to patients.

## 3. Alcohol risk Reduction for the General Population

In the general population, the justification for a discussion on alcohol risk reduction is based on the need to reduce morbidity and mortality associated with excessive alcohol consumption. In France and worldwide, data in the literature indicate a link between heavy alcohol use and the occurrence of traffic accidents, violent behaviour, injuries or problems in the workplace [4,10,11,12,13,14]. These data indicate the thresholds above which the relationship between consumption and risks can be identified (more than three standard alcoholic drinks (30 g) at one time for road accidents [13]). Apart from the societal risks, a number of psychological and physical risks have been associated with acute or chronic alcohol consumption. A direct relationship between alcohol consumption and risk of some cancers has led the French National Cancer Institute (Institut national pour la lutte contre le cancer) to discourage in 2007 regular alcohol consumption [15,16]. Similarly, a dose-dependent relationship between alcohol quantity and frequency of consumption and some liver diseases, especially cirrhosis, has been demonstrated [17]. The correlation of alcohol with some mental disorders and particularly with suicide risk is well known [18,19]. Regarding cardiovascular risks, data are more mixed and regularly cloud discussions of risk reduction related to alcohol. Indeed, even if there is a direct relationship between excessive alcohol consumption (more than five standard drinks/day) and the occurrence of vascular pathologies, moderate consumption may be more beneficial than abstinence [6,20]. This “French Paradox” is too often dragged into the promotion of alcoholic beverages and complicates the recognition of a “dose-response” effect [2]. Recent data have tended to undermine this belief, which nevertheless is deeply rooted in the French population. These data show quite clearly that “protective” alcohol (which only concerns, remember, coronary and metabolic cardiovascular risk in persons over 50 years of age) is likely a confounding issue concerning the lifestyle of people with lower cardiovascular risk [21,22]. However, apart from this particular issue, still controversial, the risk of developing an addiction, somatic disorders or impaired quality of life associated with excessive alcohol consumption is recognised and accepted by the general population. Thus, scientific authorities recommend that alcohol consumption not exceed 30 grams of pure alcohol per day (21 standard drinks per week) for men and 20 grams (14 standard drinks per week) for women outside of pregnancy [23]. It is necessary to remain very precise in the communication of this message to the general public and to specify that it is not about a definition of alcoholism but about a threshold beyond which the alcohol heavy consumption increases the risk of negative consequences on the health. Similarly, a number of preventive campaigns warn consumers not only that the threshold not be exceeded, but also advocate a return below the threshold when it is crossed, to sensitise people to the risk of the first drink [2].

Of course, to be understood by the greatest number, such messages are reduced to quantifying consumption (threshold) and do not take into account all the many and complex risk factors that determine the incidence of alcohol related diseases, starting with inter-individual vulnerability. Nevertheless, they clearly locate the alcohol risk and are part of a comprehensive strategy that, by reducing heavy consumption will reduce harms [24].

Moreover, authorities have clearly identified situational (qualitative) risks where damage may occur without crossing a threshold. These situations involve consumption associated with a particular individual risk because of vulnerability: for example, this could be pregnant women, young or old persons, people using a vehicle, a machine tool too, people who consume to relieve distress (anxiety or depression) or consumption associated with psychotropic substances [25]. Some circumstances of use are also regarded as risk situations, such as the combination of alcohol with other substances, binge drinking and especially, in the case of youngsters, repeated drunkenness. In these situations, targeted risk reduction strategies are regularly put in place by government [26]. Finally, legal measures have largely contributed to restrict access to alcohol especially for vulnerable population. Thus Evin law, adopted 11 December 1990 by the National Assembly, oversees the advertising of alcoholic beverages but does not prohibit it. Advertising is only allowed on certain media and must include a message reminding the dangers of alcohol abuse. To limit alcohol consumption, this law also forces pubs to offer their customers a sufficient choice of soft drinks.

## 4. Risk Reductions in Patients with Alcohol Use Disorders

As we have stressed, the risk reduction strategy for patients suffering from alcohol addiction must be adjusted compared with the general population. We will now focus on the alternatives, reduction of consumption *versus* permanent abstinence, and we will compare the advantages and disadvantages of these two strategies.

### 4.1. Categorical Approach versus Dimensional Approach

The traditional categorical approach, separating patients with addiction into abusers and dependents [27], has quite frequently led clinicians to consider the possibility of abusers returning to a prior “normal” use and, for addicted persons, the impossibility of finding such a consumption pattern [9]. Substance abuse concerns repeated use of alcohol despite the dangers of recurring and significant damage, whether of a family or emotional nature, social or legal, caused by the consumption [27]. For the past twenty years, under the auspices of the World Health Organization, a comprehensive effort to reduce alcohol related harm has been conducted, particularly through a program of early identification and short interventions to reduce the alcohol consumption [28]. Specific interventions designed specifically to assist people identified as being in difficulty with alcohol consumption by informing them of these difficulties (of which they were not necessarily aware), evoking the consumption risk thresholds, the alcohol content of standard drinks and the possibility of and advice for returning to a moderate level of consumption. This strategy is an example of an approach to reduce alcoholism risk.

Apart from physical signs of tolerance and withdrawal that accompany it, alcohol dependence is defined in particular by an inability to abstain from alcohol; the patient’s life is dominated by the search for alcohol and by vain attempts to avoid alcohol use [27]. Under these conditions, the strategy of risk reduction is typically associated with a total cessation of alcohol consumption [29].

This categorical approach does not allow overall quantification of the disorder and was recently supplanted by a broad definition of addiction incorporating criteria of abuse and dependence which defines stages of severity of the addictive disease [30]. According to this more comprehensive approach, one can look on the strategy of risk reduction using two approaches, abstinence *versus* reduction; these are not necessarily contradictory.

### 4.2. The Benefits of Abstinence

If we consider that alcohol dependence leads to irreversible and terminal alienation so the only way is abstinence [31]. For a long time and, for many French clinicians, mainly because of the irreversibility, the only treatment option was abstinence [31]. This approach removes the essential symptom of the disease (drinking). It is not an end in itself and accompanying psychotherapy for this “conversion or transformation” should be the rule [32]. Severance of alcohol consumption and abstinence will allow somatic, psychological and social improvements which themselves promote the extension of abstinence [33] (Table 1).

**Table 1:** The five arguments for abstinence in alcoholism.

1	Consumption reduction has not been proven as a successful strategy on a long-term basis, in particular in the scientific literature;
2	Addictive processes entail long-lasting changes in the reward circuitry (allostasis) which, even after a period of abstinence, cannot regain its previous stability;
3	This newly modified state presents a cumulating vulnerability to relapse through hypersensitisation to the smallest quantity of alcohol;
4	Sustained abstinence is the most effective strategy to stop neuronal degeneration induced by long-term alcohol consumption;
5	Abstinence remains the method of choice for the treatment of psychiatric comorbidities.

The proponents of this approach to risk reduction base their position on several arguments which we will briefly present. They argue that the reduction in consumption has not been proven as a long-term successful strategy [34]. Then, for these practitioners, neuroscience research data suggest that addictive processes are related to long lasting changes in the reward circuitry (allostasis) that, even after a period of abstinence, cannot regain its previous state of homeostasis [35]. This new modified state represents an increased vulnerability to relapse by hypersensitivity to the slightest dose of alcohol, which almost instantly triggers a return to the addictive process [36,37]. In addition, long-term abstinence is the most effective strategy for dealing with the neuronal degeneration induced by long term alcohol consumption, and therefore it is the best way to reduce the risk of developing neurodegenerative diseases related to alcohol [38,39]. Finally, abstinence remains the method of choice for treatment of psychiatric comorbidities. In most cases, when a mental disease is associated with addiction to alcohol, treatment is optimized by abstinence. In mood disorders associated with alcohol addiction, alcohol withdrawal leads to an improvement in more than 60% of cases [40].

The option of promoting abstinence in a strategy to reduce alcoholism risk is, in view of these arguments, a legitimate one and to be prioritized. Nevertheless, there are situations in which risk reduction, which must be optimal, can be achieved through the reduction of consumption.

### 4.3. The Benefits of Reduction

While this strategy might seem more appropriate in the general population in which consumption reduction is a targeted improvement in public health [41], it has a number of advantages in patients with alcohol problems. The first benefit of this strategy is to admit the maximum number of people in trouble with alcohol into the care and management system. In the United States of America, according to the Substance Abuse and Mental Health Services Administration (SAMHSA), about 21 million people in need of care for difficulties with drugs or alcohol are not treated [42]. Around 30% of cases do not enter an alcohol and drug abuse treatment program because they are not ready to meet with a caregiver who asked them to stop alcohol. Thus, a proposal of care in which reduction is a possibility, facilitates access to care by integrating the concept of a process of change into patient support and by accepting the steps involved [43]. This is plausible because a number of studies show that reducing consumption is possible during the course of the disease and is accompanied by a reduction in consequential damage [44] (Table 2).

**Table 2:** The ten arguments for consumption reduction in alcoholics.

1	The benefit obtained by the reduction of consumption corresponds to an improvement goal in terms of public health;
2	Expansion of the spectrum of available therapies is necessary to meet the demands of a larger number of patients;
3	Several Guidelines include the reduction of alcohol consumption as an “intermediate therapeutic target”;
4	Changes in the DSM-5 using a dimensional approach lead to consumption reduction as a realistic strategy in some programmes;
5	“Come back when you are motivated” is no longer an acceptable therapeutic response;
6	A goal of reducing consumption can be a first step in helping those patients who have hit a block with total abstinence to achieve success with the disease;
7	The prognosis for therapeutic success is improved if the patient chooses the goal;
8	Psychiatric comorbidities are decreased with reduction in alcohol consumption;
9	Quality of life is improved by a reduction in alcohol consumption;
10	The therapeutic approach to reducing alcohol consumption can be seen as a treatment paradigm shift.

In fact, the reduction in alcohol consumption is a risk reduction strategy that thinks otherwise about improving the patient. Patison and his team [45] were pioneers in advocating this strategy. They maintained that it would easier to improve the emotional, interpersonal and professional changes in the alcohol-dependent person by maintaining consumption (low/moderate) rather than promoting total abstinence. This method of consumption was, for Patison, a form of rehabilitation in which the patient was supported [45]. This rather revolutionary trans-Atlantic position did not find a positive response, at the time, in Europe. Twenty years later, Sobell and Sobell, turning their attention to the treatment of chronic pathologies, suggested integrating consumption reduction as a step in a therapeutic process with several stages, where, based on patient responses, the therapeutic choice can change from stage to stage [46]. Alcohol reduction is a realistic position, increasingly accepted by clinicians, which also takes account of “natural” developments for a number of patients [33]. This development, it should be recalled, is most often based on the patient’s choice. Short and medium term studies seem to suggest that the choices of total abstinence or reduced intake yield comparable results in terms of disease progression and the results are better than those of persons who do not change their consumption [47,48]. What is important is the first choice, which quite often, as an ultimate test of truth, is to reduce before stopping [49]. Hodgins reports that, based on several cohorts of patients treated for their alcohol addiction, the best marker of success was that the patient himself set the objective; it was more important than other factors such as being young, psychological and social stability, occupation and the intensity of addiction. When the planned objective is consumption reduction, it will be fully adopted by the patient and, as time goes on, redefined so as to remain realistic. Hodgins concludes that it is obvious that patients are fully capable of making good choices for themselves [50].

Thus, for many clinicians and patients, intake reduction is an unstable, but often necessary, step towards abstinence, as confirmed by subsequent longitudinal studies [51]. It can also be a palliative therapeutic strategy to support heavy drinker patients who regularly fail or are not motivated to stop drinking [33].

In addition, it is possible to think further, in particular because of recent progress in the field of drug treatment of alcoholism. Indeed the recent evaluation of the efficacy of as needed use of the opioid system modulator nalmefene in reducing alcohol consumption in patients with alcohol dependence have showed promising results on the possibility to use a treatment to allow a reduction of consumption which could continue with the long course under treatment [52].

Currently, international recommendations have included consumption reduction as a possible intermediate and supportive step leading the patient towards recovery [53,54,55]. Data on improved quality of life and improved mental conditions correlated with intake reduction clearly reinforce these recommendations [56,57]. In France, scientific authorities should start to endorse these strategies as therapies to help patients in difficulty with alcohol.

### 4.4. In Practice

Specifically and whatever the chosen strategy, the clinician should provide his patient with risk information based on current scientific knowledge and the chosen clinical approach. The pros and cons of a decision to terminate *versus* to reduce alcohol consumption must be weighed. The patient should participate in this choice and the practitioner must be lucid on the real motivational stage of the patient [58]. The possibility that the choice may change over time (a switch from one to strategy to the other) should be considered and the choice is not an end in itself, rather a means. Abstinence is the most effective strategy in terms of risk reduction, but it is not always feasible. Relapse is part of the disease and must be accepted as such; why not look on it as an attempt to control drinking? In a review combining seven multicentre studies on treatment of alcoholism, based on abstinence, Miller *et al*. have shown that 75% of participants had episodes of alcohol consumption but this could be controlled in 87% of cases [59]. A goal of reducing consumption will necessarily be based on a consumption threshold below which the patient will propose to return. For the reasons stated above, this threshold will be set by the patient. In a study of patients who had the choice of reduction versus abstinence, Adamson and Sellman have shown that among patients choosing reduction, those who had set a threshold of consumption (two drinks per day for women, three for the men) had a better prognosis than those who had a reduction strategy without a defined threshold [48].

A final practical point should be mentioned: regardless of to which patient these strategies may be proposed, there are likely to be contraindications. Amsterdam and Van den Brink recently proposed indications and contraindications for alcohol consumption reduction (Table 3). It should also be noted that, in addition to the patient’s motivation, reducing consumption requires good social integration and a supportive environment and a feeling of self-sufficiency. Or conversely, it is a strategy of “there’s no other choice.” [60].

**Table 3:** Indications and contraindications for reduced-risk alcohol consumption (from Amsterdam and van den Brink 2013).

Indications	Contraindications
Patients who have lost control and do not wish to stop drinking	Patient refusal
Suggest an early response to alcohol problems	Medical contraindications to alcohol consumption
Women, young, workers, married	CI treatments with alcohol
Mild history	Previous failure of reduction
First attempt, no involvement with AA, severe somatic or psychiatric comorbidities	Severe somatic or psychiatric comorbidities
No family history of addiction	History of severe alcohol withdrawal syndrome
Continuous consumption	
Important sense of self-efficacy	
Solid social and family stability	

Thus, the strategy of reducing consumption should aim for consumption below the recommended thresholds or, at least, in that direction. It must also be supported by pharmacotherapy and psychotherapy, which offer possibilities. Failure to manage reduction will allow the goals to be revisited and to reconsider abstinence. Finally if neither abstinence nor reduction is possible, the message should be directed at regulation of consumption and its consequences: never get behind the wheel, avoid “binges”, and avoid taking psychotropic drugs and other toxic substances simultaneously. This leads into an area well known to opiates addiction counsellors and from which experts in alcoholism may need to learn.

## 5. Conclusions

Strategies for risk reduction in alcoholism are possible. In France, they are beginning to be recognised. These strategies are different from those for consumption of illegal substances but they have similarities: different, because alcohol has a positive image and the risks associated with its use may not be widely appreciated in the general population; Similar, because alcohol can cause, in common with all other addictive substances, not only dependence but also risk starting with the first dose. We must therefore work with consumers, users at risk, abusers and addicts. The ways of supporting will differ depending on the user’s relationship with alcohol, and what it can become, but no patient should be left on the roadside.
