# Addendum: Brousse, G.; *et al.* Alcohol Risk Reduction in France: A Modernised Approach Related to Alcohol Misuse Disorders. *Int. J. Environ. Res. Public Health* 2014, *11*, 11664-11675

**DOI:** 10.3390/ijerph120505406

**Published:** 2015-05-20

**Authors:** Georges Brousse, Patrick Bendimerad, Ingrid de Chazeron, Pierre Michel Llorca, Pascal Perney, Maurice Dematteis

**Affiliations:** 1 Service Psychiatrie et Addictologie del’Adulte CMP B, Centre Hospitalier Universitaire (CHU), Rue Montalembert, Clermont-Ferrand Cedex 01 63003, France; E-Mails: idechazeron@chu-clermontferrand.fr (I.C.); pmllorca@chu-clermontferrand.fr (P.M.L.); 2 Unité de Formation et de Recherche (UFR) Médecine, Université Clermont 1, EA 7280, Place Henri Dunant, Clermont-Ferrand F-63001, France; 3 Service de Psychiatrie, Groupe Hospitalier La Rochelle- Ré-Aunis, La Rochelle 17000, France; E-Mail: patrick.bendimerad@free.fr; 4 Service de Addictologie, Hôpital Caremeau, Nîmes F-30029, France; E-Mail: pascal.PERNEY@chu-nimes.fr; 5 Université de Montpellier 1, 5 bd, Henri IV 34000, France; 6 University of Grenoble Alpes, HP2, F-38000 Grenoble, France; E-Mail: MDematteis@chu-grenoble.fr; 7 INSERM, HP2, U1042, F-38000 Grenoble, France; 8 Department of Addiction Medicine, CHU de Grenoble, HP2, F-38000 Grenoble, France

The author would like to update “Conflicts of Interest” section of their previous publication [1] as follows:
